# MicroRNA Stability in FFPE Tissue Samples: Dependence on GC Content

**DOI:** 10.1371/journal.pone.0163125

**Published:** 2016-09-20

**Authors:** Yu Kakimoto, Masayuki Tanaka, Hiroshi Kamiguchi, Eriko Ochiai, Motoki Osawa

**Affiliations:** 1 Department of Forensic Medicine, Tokai University School of Medicine, Isehara, Kanagawa, Japan; 2 Support Center for Medical Research and Education, Tokai University, Isehara, Kanagawa, Japan; University of Massachusetts Medical School, UNITED STATES

## Abstract

MicroRNAs (miRNAs) are small non-coding RNAs responsible for fine-tuning of gene expression at post-transcriptional level. The alterations in miRNA expression levels profoundly affect human health and often lead to the development of severe diseases. Currently, high throughput analyses, such as microarray and deep sequencing, are performed in order to identify miRNA biomarkers, using archival patient tissue samples. MiRNAs are more robust than longer RNAs, and resistant to extreme temperatures, pH, and formalin-fixed paraffin-embedding (FFPE) process. Here, we have compared the stability of miRNAs in FFPE cardiac tissues using next-generation sequencing. The mode read length in FFPE samples was 11 nucleotides (nt), while that in the matched frozen samples was 22 nt. Although the read counts were increased 1.7-fold in FFPE samples, compared with those in the frozen samples, the average miRNA mapping rate decreased from 32.0% to 9.4%. These results indicate that, in addition to the fragmentation of longer RNAs, miRNAs are to some extent degraded in FFPE tissues as well. The expression profiles of total miRNAs in two groups were highly correlated (0.88 <*r* < 0.92). However, the relative read count of each miRNA was different depending on the GC content (*p*<0.0001). The unequal degradation of each miRNA affected the abundance ranking in the library, and miR-133a was shown to be the most abundant in FFPE cardiac tissues instead of miR-1, which was predominant before fixation. Subsequent quantitative PCR (qPCR) analyses revealed that miRNAs with GC content of less than 40% are more degraded than GC-rich miRNAs (*p*<0.0001). We showed that deep sequencing data obtained using FFPE samples cannot be directly compared with that of fresh frozen samples. The combination of miRNA deep sequencing and other quantitative analyses, such as qPCR, may improve the utility of archival FFPE tissue samples.

## Introduction

MicroRNAs (miRNAs) are essential regulators of biological activity, suppressing gene expression at the posttranscriptional level [1]. MiRNA expression alterations profoundly affect cell differentiation and metabolic activity, and they are often involved in the pathogenesis of human diseases [2–4]. Currently, miRNA quantitative analyses, including microarray, deep sequencing, and quantitative PCR (qPCR), are extensively performed, using different types of patients’ tissues.

Formalin-fixed paraffin-embedded (FFPE) tissue samples are valuable research materials, stored in pathology archives worldwide. Storing FFPE specimens is more economical than storing frozen samples, and the histological structure can be preserved almost permanently. However, formalin fixation and paraffin embedding inevitably lead to nucleic acids degradation in these tissues DNA and RNA are fragmented and chemically modified [5,6], which sometimes causes inconsistency between the results obtained using matched fresh or FFPE samples in genotyping and other quantitative analyses [7].

Compared with longer RNA and DNA molecules, small RNAs, including miRNAs, are very robust and resistant to severe changes in pH or temperature, as well as the repeated ice/thaw cycles [8,9]. We showed previously that miRNAs are stably detected in postmortem tissue samples and that qPCR analysis can be performed even after prolonged formalin fixation [10]. Furthermore, miRNA deep sequencing has recently been performed using FFPE specimens, and good overall correlation with the results obtained by analyzing the matched frozen samples has been reported [11–14]. However, the expression levels of the individual miRNAs in the matched frozen and FFPE samples were not precisely assessed in these studies, which could represent a critical pitfall during the identification of specific disease biomarkers using archival FFPE samples.

Here, we quantified cardiac miRNAs in matched frozen-FFPE tissue samples using next-generation sequencing. The comparison between matched samples showed considerable discrepancy in the expression levels of several miRNAs, even though total miRNA expression signatures were strongly correlated. Subsequent qPCR analyses demonstrated a close relationship between the degradation level and the GC content of miRNAs in FFPE specimens. This study provides a novel insight into the miRNA stability in FFPE tissue samples, and promotes further utilization of stored tissue samples in the comprehensive analyses of miRNAs.

## Materials and Methods

### Tissue sampling and histological analyses

Human cardiac tissue samples were obtained at autopsy. A part of the left ventricular free wall was immediately immersed in liquid nitrogen and stored at -80°C for RNA analyses. Serial parts of the cardiac tissue were underwent FFPE process, and were microscopically examined. No apparent histopathological changes were observed in these study samples.

The cadavers were stored at 4°C before the autopsy, and the interval between death and tissue sampling ranged from 20 to 120 h. FFPE specimens underwent 10% formalin fixation at room temperature for 5 days to 1.5 months. Afterward, the fixed samples were washed with water for 12 h, followed by washing with ethanol (70–100%) six times, and with xylene three times. The dehydrated tissues were embedded in paraffin at 62°C, and stored at room temperature. Total RNA was isolated from frozen samples within 2 months after the autopsy, and from FFPE samples within 13 months after paraffin embedding.

A total of 10 paired frozen and FFPE samples were analyzed. The patients’ characteristics and sample storage conditions are summarized in S1 Table. The study protocol and all experimental procedures were approved by the ethics committee of Tokai University (14I-06), and the study has been conducted according to the principles expressed in the Declaration of Helsinki. Written informed consent allowing the experimental use of the tissue samples was obtained from the bereaved relatives of all patients.

### RNA isolation

RNase inhibitor was sprayed on the workbench before handling the samples, and RNase-free water and equipment were used for all miRNA analyses. Total RNA isolation and subsequent small-size RNA selection from the frozen tissue samples were performed using mirVana miRNA Isolation Kit (Applied Biosystems, Foster City, CA, USA), according to the manufacturer’s protocol. Purified RNA was eluted with 100 μL of 95°C Elution Solution, from approximately 100 mg of crushed tissue.

FFPE tissue samples were cut into 20-μm thick sections, and one or two of these sections were used for total RNA isolation with the RecoverAll Total Nucleic Acid Isolation Kit (Applied Biosystems), according to the manufacturer’s protocol. The tissue was immersed in xylene and deparaffinized at 50°C for 5 min. After two washes with ethanol and subsequent centrifugation, the pellet was vacuum-dried. Protease digestion was performed overnight at 50°C, and DNase digestion was performed for 30 min at room temperature. Finally, RNA was eluted with 40 μL of Elution Solution.

RNA concentration and purity were measured using a spectrophotometer (BioSpec-nano; Shimadzu, Kyoto, Japan). RNA purity and integrity were assessed using microcapillary electrophoresis, which was performed on a 2100 Bioanalyzer with Small RNA kit (Agilent Technologies, Santa Clara, CA, USA). All RNA samples were stored at -80°C until further processing.

### MiRNA deep sequencing and data processing

Small RNA library was prepared from 24 ng of miRNA obtained from each cardiac sample, using the Ion Total RNA-seq kit v2 (Life Technologies, Carlsbad, CA, USA), according to the manufacturer’s instructions. Briefly, 3′ and 5′ adapters were hybridized to small RNAs, which was followed by an overnight ligation. After the reverse transcription, complementary DNA molecules (cDNAs) were purified and size-selected using magnetic beads. Afterward, cDNA samples were PCR-amplified using a common primer set. No barcode was added, because we sequenced only one library per chip, in order to gain the maximum read depth. The amplicons were purified again and size-selected with magnetic beads. Final yields and size distribution of the amplified cDNAs were assessed by 2100 Bioanalyzer with a High Sensitivity DNA kit (Agilent Technologies).

Subsequently, 5 μL of each library (100 pmol/L) were sequenced on an Ion 318 chip using the Ion Torrent PGM system (Life Technologies). Sequencing data was transferred to the Torrent Browser, where the adapter sequences were removed and low quality reads, such as primer dimers, were excluded. Following this, the filtered reads were successively mapped to the UCSC human genome hg19, using TMAP program (Life Technologies), and to the human miRNA database, miRBase v21, using SHRiMP2 program [15], with default parameters. The coverage depth data were analyzed using CLC Genomics Workbench v6.0.1 (QIAGEN, Venlo, Netherlands).

The read counts of each known miRNA were normalized to the library size (total number of generated reads) and presented as reads per million mapped (RPM). Only miRNAs with normalized read counts over 10 in at least one sample were further analyzed. Correlation was tested using Pearson’s correlation coefficient. The GC% of mature miRNAs was compared between the decreased (<0.5-fold) and increased (>2-fold)/unchanged group using Dunnett’s test, and *p*<0.01 was considered statistically significant.

### RT-qPCR

To validate the results of miRNA deep sequencing, we selected seven target miRNAs, which were detected in the cardiac tissue in abundance. All of them are 22 nucleotides long, and their GC content (GC%) was shown to range from 27% to 64%. The target sequence data are shown in Table 1.

**Table 1:** Target miRNA characteristics.

miRBase ID	TaqMan ID	Sequence	Length (nt)	GC (nt)	GC (%)
hsa-miR-99b-5p	000436	CACCCGUAGAACCGACCUUGCG	22	14	64
hsa-miR-133a-3p	002246	UUUGGUCCCCUUCAACCAGCUG	22	12	55
hsa-miR-133b	002247	UUUGGUCCCCUUCAACCAGCUA	22	11	50
hsa-miR-22-3p	000398	AAGCUGCCAGUUGAAGAACUGU	22	10	45
hsa-let-7e-5p	002406	UGAGGUAGGAGGUUGUAUAGUU	22	9	41
hsa-miR-21-5p	000397	UAGCUUAUCAGACUGAUGUUGA	22	8	36
hsa-miR-1-3p	002222	UGGAAUGUAAAGAAGUAUGUAU	22	6	27

cDNA was synthesized from 5 ng of total RNA using a TaqMan MicroRNA Reverse Transcription kit (Applied Biosystems), according to the manufacturer’s instructions. All cDNA samples were stored at -20°C until real-time PCR analyses. Each primer set was used with no template as well, and the synthesized product was used as a negative control in the subsequent analyses.

Mature miRNAs were quantified using a StepOnePlus real-time PCR system (Applied Biosystems). Serially diluted cDNAs were used as the template for calculating PCR efficiency for each primer set. Data processing was performed using StepOnes^TM^ software, version 2.3 (Applied Biosystems). The threshold value was set at 0.02 throughout the study. PCR reactions were performed according to the MIQE (minimum information for publication of quantitative real-time PCR) guidelines [16], where applicable.

Triplicate Ct values obtained for 20-fold diluted cDNA were averaged, and the relative expression of target miRNAs was determined by the ΔΔCt method [17]. Comparisons between the changed/unchanged groups were performed with Mann Whitney *U*-test, and *p*<0.01 was considered statistically significant.

## Results

### Read count and read length

**Fig 1:**
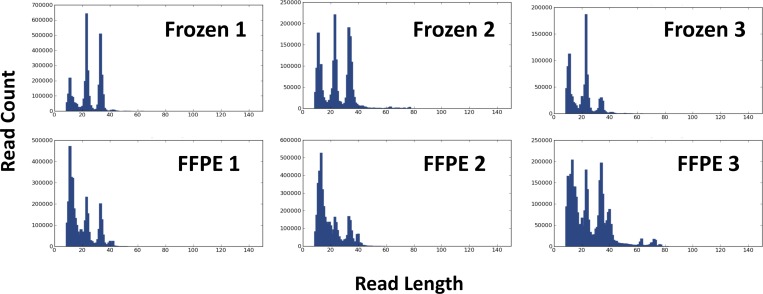
Read length histograms obtained from the matched frozen and FFPE samples. All samples showed three prominent peaks. The most frequent read length was 22 nt in frozen samples, and 11 nt in FFPE samples.

Matched frozen and FFPE samples obtained from three normal hearts were used for miRNA sequencing. After trimming the adaptor sequences from the sequenced reads, the most frequent read length was shown to be 22 nt and 11 nt in frozen and FFPE samples, respectively (Fig 1).

Of the total 6,462,853 reads that were generated from the three frozen samples, 5,979,759 reads (92.1%) were aligned to the human genome sequence (hg19), and 2,080,072 of these reads (32.0%) were mapped to the known miRNAs (miRBase v21).

In contrast, of the total 11,388,982 reads generated from three FFPE samples, 9,905,331 reads (87.0%) were aligned to the human genome sequence, among which 1,070,701 (9.4%) were mapped to the known miRNAs. Sample FFPE 2, with the longest fixation period, showed the lowest mapping rate (5.6%; Table 2).

**Table 2:** MiRNA deep sequencing results.

Sample ID	Frozen 1	Frozen 2	Frozen 3	FFPE 1	FFPE 2	FFPE 3
**Mode length (nt)**	22	22	22	10	12	12
**Total reads**	3,438,923	2,061,964	991,966	3,563,818	4,416,616	3,408,548
**Mapped reads**	3,222,047	1,860,747	896,965	3,236,721	3,693,029	2,975,581
**Percent reads on hg 19 (%)**	94	90	90	91	84	87
**Percent reads on miRBase v21 (%)**	36	24	35	14	6	9

In total, 1,046 mature miRNA species were sequenced from six libraries used in this study (doi:10.5061/dryad.fj0f8, S2 Table).

**Fig 2:**
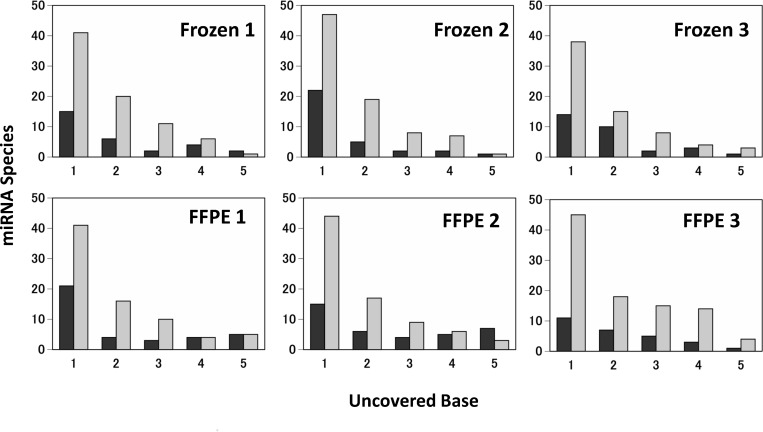
Fragmentation at miRNA 5’ and 3’ ends. Black bar indicates 5’ end, and gray bar indicates 3’ end. The degradation occurred more at the 3’ end than the 5’ end, and longer sequences were more frequently lost in FFPE samples than in frozen samples.

Among the mapped miRNAs, some fragmentation was observed at both ends (Fig 2). More bases were lost at the 3’ end than that the 5’ end. Furthermore, more bases were lost in the FFPE samples than in the frozen samples.

### Deep sequencing and GC%

**Fig 3:**
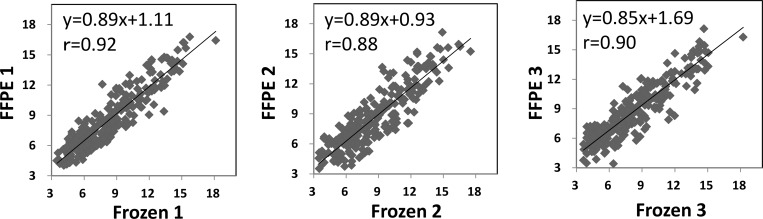
Scatter plots of sequenced miRNAs in the matched frozen and FFPE samples. Log_2_ scales are used for both axes (normalized read counts of 240 miRNAs). Linear regression and the appropriate equations are indicated.

We focused on 240 miRNAs that were represented by >10 RPM in at least one library, and compared the normalized read count of each miRNA between the matched frozen and FFPE samples. The three paired samples showed highly correlated total miRNA logarithmic expression levels, with *r* ranging from 0.88 to 0.92 (Fig 3).

**Fig 4:**
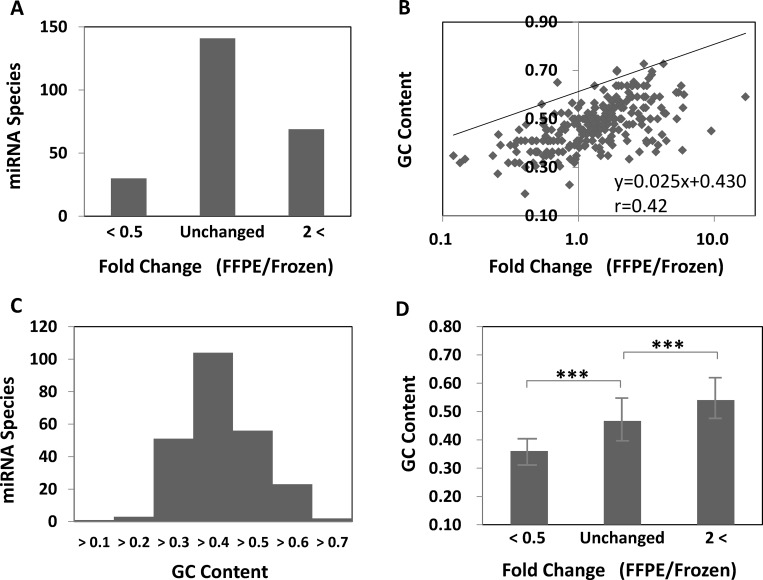
MiRNA read count and GC content in matched frozen and FFPE samples. (A) Levels of 30 miRNAs decreased, and those of 69 miRNAs increased in FFPE samples compared with frozen samples. (B) The miRNA variability between the matched frozen-FFPE samples was moderately correlated with the GC content of the miRNAs. (C) The GC% in 240 miRNAs ranged from 19% to 73%, and the average GC% was shown to be 48 ± 10%. (D) The miRNAs with unchanged levels had moderate GC% (47 ± 8%). The miRNAs with decreased levels showed lower GC% (36 ± 6%), and miRNAs with higher levels had higher GC% (54 ± 9%). The GC content was calculated based on the mature miRNA sequence. Data are presented as mean ± SD; *** *p*<0.0001.

However, at the individual miRNA levels, some values were shown to differ between the fresh and FFPE samples. The expression levels of 30 miRNAs decreased to less than 50%, and the levels of 69 miRNAs increased more than 2-fold in FFPE samples compared with frozen samples (Fig 4A). The miRNA variance between the matched frozen-FFPE samples was shown to be moderately correlated with the GC content of miRNAs (Fig 4B).

GC% in 240 investigated miRNAs ranged from 19% (miR-590-3p; 4nt/21nt) to 73% (miR-887-3p and miR-1307-3p; 16nt/22nt), and the average GC% was shown to be 48 ± 10% (mean ± standard deviation (SD), Fig 4C). We then divided the 240 miRNAs into 3 groups: those in which the miRNA levels decreased to less than 50% of the original level, those in which the miRNA levels increased by more than 2-fold, and those in which the miRNA levels were unchanged. The GC% of the miRNAs whose expression levels did not change between fresh and FFPE samples, was 47 ± 8% (Fig 4D). In contrast to this, miRNAs whose levels decreased, had lower GC contents (36 ± 6%, *p* = 3.2×10^−8^), while miRNAs whose expression increased were shown to have higher GC% (54 ± 9%, *p* = 4.3×10^−8^). Therefore, miRNA stability in FFPE samples was shown to be strongly related to its GC content.

### MiRNA ranking as obtained by deep sequencing analysis

**Fig 5:**
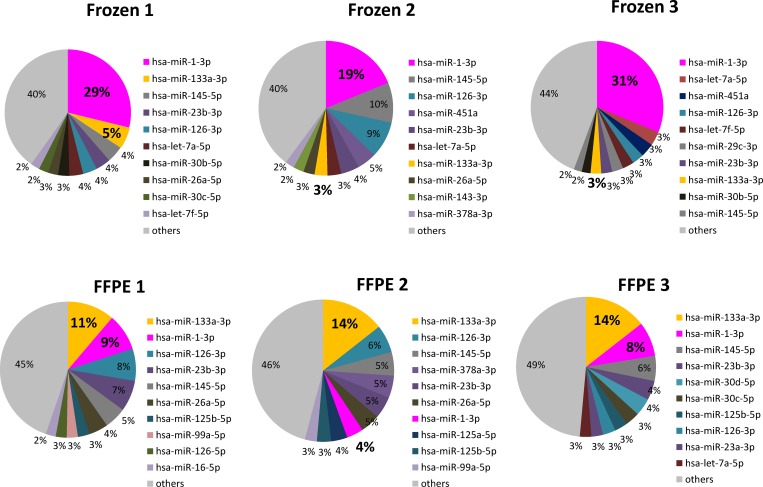
Read count ranking of cardiac miRNAs. All samples contained similar abundant miRNA species. However the ranking of each miRNA was different in the matched frozen and FFPE samples.

Ten most abundant miRNAs in each library are shown in Fig 5, and they represent more than 50% of the total miRNA read counts in each sample. The major miRNA species in FFPE tissues were almost the same as those found in frozen tissues. However, some miRNA levels differed between the matched samples. For example, the most abundant miRNA in frozen sample was miR-1-3p, which accounted for 20–30% of all read counts. In contrast to this, the main miRNA found in FFPE tissues was miR-133a-3p, which was shown to represent up to 14% of total miRNAs, while miR-1-3p represented no more than 10% of total miRNAs in FFPE samples.

### qPCR and GC% analysis

**Fig 6:**
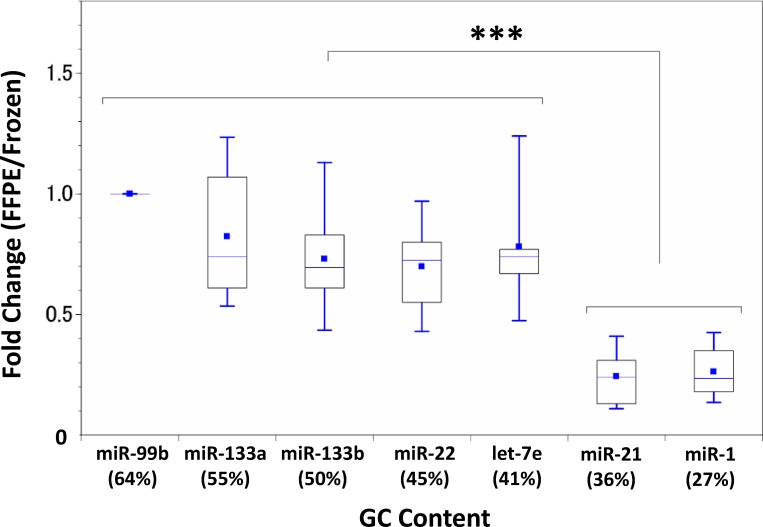
GC content and qPCR analysis of miRNA in matched frozen and FFPE samples. MiR-99b-5p was used as an internal control. In FFPE samples, miRNAs with higher GC% (>40%) showed less of a decrease (0.81 ± 0.25) than miRNAs with lower GC% (0.25 ± 0.11). n = 10 pairs *** *p*<0.0001.

In order to confirm the influence of GC% on miRNA stability in FFPE specimens, we performed qPCR analysis of seven miRNAs with GC% of 27–64% (Table 1), with miR-99b-5p (GC 64%) as internal control. Standard curves for each primer set showed amplification efficiency of 100 ± 10% (S3 Table). We demonstrated that miRNAs with higher GC% were preserved better in FFPE samples (Fig 6, S4 Table). Additionally, miRNAs with GC% of less than 40% were significantly degenerated in FFPE specimens (*p* = 1.4×10^−10^).

## Discussion

### MiRNA stability in FFPE samples

Formalin fixation leads to RNA degradation, which often affects subsequent quantitative analyses [5–7]. In this study, the average read length of RNAs in FFPE tissues was shown to be shorter than that in the matched frozen samples, while the total rate of their mapping to the human genome was not considerably changed in FFPE samples. The obtained results indicate that longer RNAs were fragmented into smaller RNAs, which led to an increase in the total read counts in FFPE samples. Additionally, the decrease in miRNA mapping rates and the increase of uncovered bases of miRNAs imply that, similar to longer RNAs, miRNAs are degraded in FFPE specimens to some extent as well. Interestingly, the degradation occurred more in the 3’ end than 5’ end. Several miRNA-degrading enzymes have been identified, including both 3’-to-5’ and 5’-to-3’ exoribonucleases, but thus far no endoribonucleases. The activity of the exoribonucleases may be different between both ends. Moreover, the lack of a poly-A tail at the 3’ end of miRNAs may induce degradation after tissue sampling. However, the exact mechanism of miRNA turnover remains largely unknown.

During tissue sample handling and long-term storage, environmental conditions, including temperature change and UV radiation, can promote RNA degradation [18, 19]. We have previously demonstrated that the read length of RNA is strongly related with its stability in FFPE samples, and miRNAs are more abundantly detected than longer RNAs after prolonged fixation [10]. Here, we have focused on the difference in the stability among miRNAs in FFPE specimens. Deep sequencing and qPCR analyses demonstrated that guanine (G) and cytosine (C) content influences the stability of the miRNAs in the formalin fixed samples.

RNA stability *in vivo* is controlled through the binding of RNA degrading enzymes to the specific sequences or secondary structures [20]. High GC% at the third codon position and GC alleles of single nucleotide polymorphisms (SNPs) were shown to enhance mRNA stability in mammalian cells [21,22]. RNA levels are well balanced by the transcription and RNA degradation *in vivo*, while the RNA abundance in stored tissue samples is determined mainly by RNA decay rate. Although the RNA destruction process in FFPE tissue is different from that *in vivo*, mRNAs with higher GC% were reported to be less affected by FFPE degradation than GC-poor mRNAs [23]. Taken together, these results show that higher GC% allows higher miRNA and mRNA stability in archival FFPE tissues.

### Global normalization during the deep sequencing of miRNA

Normalization process allows the comparisons of gene expression levels between different samples in RNA quantitative analysis. The simplest and least invasive approach for RNA deep sequencing is the normalization of the total number of reads in each library, which provides the relative ranking of individual miRNA expression levels [24,25]. Presenting each expression level as reads per kilobase per million mapped reads (RPKM) represents a commonly used normalization method [26–28]. Here, we used RPM instead of RPKM, because all miRNA were 18–24 nucleotides long, and the difference in the read length is assumed to have little influence on the read depth during miRNA sequencing [29–31]. As these sum-based normalization processes postulate constant total read count in every library, upon the decrease of a single read counts, the other miRNA read counts increase. This may present a serious issue for miRNA deep sequencing using FFPE tissues, where the degradation level of each miRNA is different. For example, the significant decrease in miR-1-3p levels, which was shown to be the dominant miRNA in cardiac muscle before the fixation, led to the increase in the read counts of other miRNA in FFPE samples. The normalization of the total read counts exaggerates the difference in the relative expression levels of miRNAs, during the deep sequencing analysis of FFPE specimens.

### FFPE sample utility in miRNA analysis

Our data demonstrated some limitations of FFPE sample utilization for miRNA deep sequencing. As the degradation speed varies considerably among miRNAs, the relative expression level of each miRNA in FFPE tissue may differ from that in the matched frozen tissue. Therefore, miRNA ranking by abundance differed between the paired samples in this study. This discrepancy shows that the read depth of each miRNA in FFPE sample cannot be directly compared with that in frozen samples, even after the normalization.

To date, different library preparations, sequencing platforms, and mapping algorithms were shown to affect the results of RNA deep sequencing [32–34]. For example, the results showing that miR-1 represents the most abundant miRNA in human cardiac tissues are consistent with the results previously obtained, but the subsequent ranked miRNAs were not perfectly matched [35,36]. Additionally, a different sequencing study reported that miR-143 is the most abundant miRNA in human heart tissue [37]. Therefore, assessment of the absolute copy number of transcripts based only on the sequencing data is difficult.

In contrast, qPCR represents a reliable quantitative method even for FFPE specimen. Little difference was observed among qPCR protocols with constant PCR efficiency. qPCR are generally normalized using endogenous controls [16], and the differences in the stability of miRNA species do not considerably affect the quantification under the same fixation/storage conditions. Although the relative abundance of each miRNA is unavoidably altered by the fixation process, the change in the expression of the target miRNA in FFPE samples is more stably quantified by qPCR than by deep sequencing. However, the limited number of miRNAs that can be quantified at the same time represents a practical disadvantage of qPCR.

Deep sequencing approach represents a high throughput analysis, which is quite attractive as a screening tool for biomarker discovery using patients’ tissues. Combining deep sequencing and qPCR validation should allow the comprehensive analyses of miRNA using FFPE tissue specimens.

### Limitations

In this study, we compared matched frozen and FFPE tissues and considered that the quantitative difference between the two groups was mainly dependent on the preservation condition after sampling. However, the difference between the groups could also have been due to the RNA extraction method, including deparaffinization. As different extraction protocols are required for frozen and FFPE specimens, it is possible sample handling from preservation to RNA extraction could affect library preparation and sequencing efficiency.

Cardiac disease is the major cause of sudden natural death; therefore, it is important to determine the stability of biomolecules to facilitate the diagnosis of diseases in human cardiac tissues obtained at autopsy and preserved using various methods. As we only used cardiac tissue in this study, the stability of miRNAs determined here might be different from that in other tissue types.

## Conclusions

This is the first study to demonstrate the differences in the stability of miRNAs in FFPE samples. GC-poor miRNAs (GC<40%) were shown to be more degraded than GC-rich miRNAs. Although miRNAs are more robust than mRNAs, the deep sequencing data obtained using FFPE samples cannot be directly compared with the data obtained using fresh frozen tissue samples. The miRNA read counts in FFPE specimens are comparable only if the samples are prepared under the constant fixation conditions, and using the same sequencing protocol. For accurate quantification, miRNA deep sequencing using FFPE tissue has to be accompanied by other quantitative analyses, including qPCR validation. Multiple quantitative approaches may broaden the utility of archival FFPE samples in the future.

## Supporting Information

S1 Table Patient characteristics and sample storage conditions. (XLSX)

S2 Table Cardiac miRNAs with at least one sequenced sample. (XLSX)

S3 Table Cardiac miRNA qPCR standard curves. (XLSX)

S4 Table Ct values obtained by cardiac miRNA qPCR. (XLSX)
